# *In silico* EST-SSR Identification and Development through EST Sequences from *Metroxylon sagu* Rottb. for Genetic Diversity Analysis

**DOI:** 10.21315/tlsr2024.35.1.2

**Published:** 2024-03-30

**Authors:** Devit Purwoko, Siti Zulaeha, Teuku Tajuddin, Farida Rosana Mira, Maharani Dewi Solikhah, Gemilang Rahmadara, Nurul Fitri Hanifah, Rusmanto

**Affiliations:** 1 Research Centre for Applied Botany, Research Organization for Life Sciences and Environment, National Research and Innovation Agency, Science and Technology Park of Soekarno, Cibinong, Bogor West Java 16911, Indonesia; 2 Directorate of Laboratory Management, Building 630, Science and Technology Park of B. J. Habibie, Serpong, South Tangerang 15314, Indonesia; 3 Research Centre for Energy Convention and Conservation, National Research and Innovation Agency, Science and Technology Park of B.J. Habibie, Serpong, South Tangerang 15314, Indonesia

**Keywords:** EST, *in silico*, *Metroxylon sagu*, SSR, Genetic Diversity

## Abstract

Sago plant (*Metroxylon sagu* Rottb.) is one of the most carbohydrate-producing plants in the world. Microsatellites or simple sequence repeats (SSRs) play an important role in the genome and are used extensively compared to other molecular markers. For the first time, we are exploiting data expressed sequence tags (EST) of sago plants to identify and characterise markers in this species. EST data about sago plants are obtained through the EST database on the National Center for Biotechnology Information (NCBI) website. We obtained data of 458 Kb (412 contig) with a maximum and minimum length of 1,138 and 124 nucleotides, respectively. We successfully identified 820 perfectly patterned SSR using Phobos 3.3.12 software. The type characterisation of EST-SSR was dominated by tri-nucleotides 36% (294), followed by hexa-nucleotides 24% (202), tetra-nucleotides 15% (120), penta-nucleotides 13% (108) and di-nucleotides 12% (96). The most frequency of SSR motifs in each type is AG, AAG and AAAG. Analysis of synteny on the EST sequence with the online application Phytozome found that sequences were distributed on 12 *Oryza sativa* chromosomes with a likeness percentage between 63% to 100% and e-value between 0 to 0.094. We developed the primer and generated 19 primers. Furthermore, we validated 7 primers that all generated polymorphic alleles. To our knowledge, this report is the first identification and characterisation of EST-SSR for sago species and these markers can be used for genetic diversity analysis, marker assisted selection (MAS), cultivar identification, kinship analysis and genetic mapping analysis.

**Highlights**

- A computational-based approach was used to develop and identify simple sequence repeat (SSR) markers from a publicly available expressed sequence tags (EST) database.
- The identification, characterisation and development of EST-SSR markers can be used for genetic diversity analysis, marker assisted selection (MAS), cultivar identification, kinship analysis and genetic mapping analysis.
- New EST-SSR markers were successful used for genetic diversity analysis of sago palm.

## INTRODUCTION

Genetic diversity among individuals or populations is the basis of adaptation and evolution and thus plays a major role in dealing with different biotic and abiotic pressures. Diverse genetic resources provide better opportunities for plant breeders to create new, better cultivars with desired traits (Salgotra & Chauhan 2023). The study of genetic diversity in plant species is very useful for the development of breeding programmes and for conservation purposes. To access genetic variation within and between populations, morphological characterisation approaches, biochemical markers and molecular markers are often used (Mondini *et al*. 2009). Assessment of variation between populations using morphological characters is difficult to study because morphology varies under different plant growth conditions (D’Imperio *et al*. 2011). DNA markers are critical for assessing genetic diversity between and within different plant species (Amiteye 2021; Hailu & Asfere 2020), because they highlight differences in nucleotide sequences between individuals and are not affected by environmental factors (Aslanbay Guler & Imamoglu 2023).

Simple sequence repeat (SSR) genetic markers are currently widely used for genetic diversity analysis, cultivar identification, pedigree analysis, genetic mapping analysis and marker-assisted selection (MAS). SSR markers, also known as microsatellite markers, if used as genetic markers, are codominant, polymorphic so that they have a high level of allele diversity, and the test is very efficient because it is based on the Polymerase chain reaction (PCR) method (Molla *et al*. 2010). Therefore, SSR markers can be used to detect the diversity among closely related plant accessions better than other molecular markers (Kumar *et al*. 2009). SSR markers can be developed through genomic and genic approaches.

SSR markers with a genomic approach (g-SSR) were developed through the identification of genomic sequences while SSR markers with a genic approach (EST-SSR) were developed through expressed sequence tags (EST) sequences (Jain *et al*. 2014). The development of SSR markers using traditional methods requires a lot of time, money, and laboratory work (Kale *et al*. 2012). The development of SSR markers from genomic libraries will be time-consuming and require large infrastructure laboratory facilities. An alternative and more effective approach that can be done is searching for SSR *in silico* in the EST database that has been published in NCBI. This method has been widely used in various molecular studies on various plants (Purwoko *et al*. 2021; Priyanka *et al*. 2017; Vieira *et al*. 2016; Jain *et al*. 2014; Aberlenc-Bertossi *et al*. 2014; Duran *et al*. 2013).

Sago (*Metroxylon sagu* Rottb.) is one of the palm plants that produce starch. Sago plants can accumulate starch in the trunk up to 200 kg/tree to 220 kg/tree (Jong 1995) and are one of the high carbohydrate-producing plants in the world (Flach 1995). The utilisation of sago is very dependent on the potential of available sago resources. Uncontrolled exploitation of sago is carried out to fulfill the need for food, industrial raw materials and energy which continues to increase, causing productive sago palm species to be threatened with extinction. One way to protect Indonesian germplasm, especially sago palms, is to conduct an inventory and characterisation both phenotypically and genotypically. Genetic markers are known to have an important role in uncovering and studying plant diversity and population genetics with techniques to detect genetic variability between individuals, populations and species. Knowledge of genetic variability is a prerequisite for studying the evolutionary history of a species and also for breeding programs and conservation of plant genetic resources. Data on genetic diversity is needed to protect sago palms and their genetic components, which are thought to be native to Indonesia, from being exploited by other countries.

Recently, SSR markers have been developed using partial genome data to study the genetic diversity of sago palms (Purwoko *et al*. 2019) but the development of SSRs using EST data for sago palms has not been fully studied. Sago EST data have been generated and published in a publicly accessible database offering the opportunity to create EST-SSR markers *in silico*. This approach can be used to design specific primers at specific loci that represent functional genes or coding regions. The development of SSR markers using EST sequences has several advantages compared to genomic sequences, such as EST-SSR represents functional components of the genome and can be used between species, can be used to search for genes, and also map genes. Identification of SSR through these two approaches has been widely carried out on palm trees such as oil palm and dates. So far, the identification of SSR in sago palm with the above approach has not been reported. This is the first report of the SSR analysis of sago palms using the genic approach (EST-SSR).

## MATERIAL AND METHODS

### Plant Material

**Figure 1:**
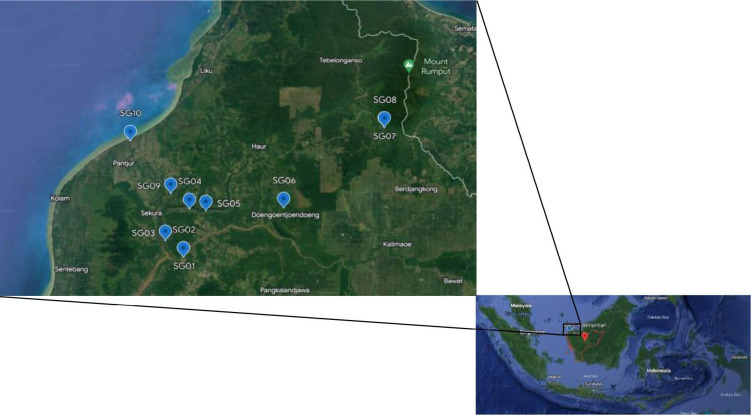
Coordinate map the origin West Borneo of sago palm samples used to analyse the genetic diversity using EST-SSR markers. (Source: Google Earth Engine).

Plant material from 10 accessions of *Metroxylon sagu* Rottb. (leaves) were collected from various regions in West Kalimantan (Fig. 1) along with three other accessions: 1 accession from Java (S1), 1 accession from Sumatra (B1), and 1 accession from Maluku (C4).

### EST Sequence Source, SSR Analysis and Functional Characterisation

The EST sequence of sago palm was obtained from the NCBI database (http://ncbi.nlm.nih.gov/) with accession number JK731189-JK731600 which is the EST of young leaves of the sago palm. The EST sequences that have been downloaded from the NCBI website are then uploaded to the EGassembler website (https://www.genome.jp/tools/egassembler/) which aims to clean sequences, remove vector contamination, and assemble contig sequences (Nejad *et al*. 2006). Sequence processing is carried out using standard parameters suggested by the site. Contig sequences resulting from the assembly and processing were then downloaded in FASTA format for further use for SSR analysis, synthesis and functional gene analysis.

SSR analysis was performed using Phobos 3.3.12 software (Mayer *et al*. 2010) to detect nucleotides at loci with di-, tri-, tetra-, penta- and hexa-nucleotide motifs. Synteny analysis was carried out using the Phytozome online application using the BLASTn programme. From the EST sequences that were detected to have SSR and were selected (at least 20 bp lengths of SSR), synteny analysis was carried out using *Oryza sativa* chromosome data. The EST sequence containing the SSR motif was then searched for putative genes by comparing the non-redundant protein *Arabidopsis* database on The *Arabidopsis* Information Resource (TAIR) (http://www.arabidopsis.org/index.jsp) using the BLASTx programme with an e-value limit of 10^−3^. The gene ontology (GO) mapping analysis aims to provide annotations of the highest BLAST Hit results (Gotz *et al*. 2008). After mapping GO, then proceed with GO annotation which aims to provide functional annotations to query sequences (Gotz *et al*. 2008). The parameters used for GO annotation include annotation cut off of 55, GO weight of 5, Hit-filter e-value of 1.0^e-6^, and HSP-Hit coverage cut off of 0. Visualisation of GO analysis results using the http web-based REVIGO application (http://revigo.irb.hr/Results.aspx?jobid=738236493) (Supek *et al*. 2011).

### Total DNA Isolation, Primer Design and Validation

After the motifs and synteny analysis results were obtained, the primers were designed using Primer3 1.1.4 software (Untergasser *et al*. 2012). The parameters used for the primary design are presented in Table 1.

Total DNA was isolated from sago leaf samples using a modified cetyltrimethylammonium bromide (CTAB) method for DNA isolation from palm leaves (Purwoko *et al*. 2019; Maskromo *et al*. 2016; Novero *et al*. 2012; Pesik *et al*. 2015; 2017; Tinche *et al*. 2014). To check the quality and concentration of DNA, electrophoresis was used with 1% agarose gel. The PCR composition was made with a total volume of 25 μL/reaction consisting of Go taq green master mix (12.5 μL), forward primer (2 μL), reverse primer (2 μL), 2 μL DNA template and sterile H_2_O to a volume of 25 μL. Takara PCR Thermal Cycler Dice^®^ (http://catalog.takara-bio.co.jp/product/basic_info.php?unitid=U100004192) was used for amplification of SSR markers. The PCR program used was as follows: predenaturation at 95°C for 3 min, denaturation at 95°C for 30 s 35 cycles, annealing with Tm − 5°C for 30 s and extension at 72°C for 30 s each for 35 cycles and final extension 72°C for 60 s and hold at 4°C. The PCR products were evaluated using gel electrophoresis in 1% agarose and finally visualised with SYBR safe dye (Invitrogen). The amplified product which is expected to be of band size is further separated on the 8% agarose metaphor gel.

### Genetic Analysis

Using a straightforward matching dissimilarity index, we created a dissimilarity matrix for the diploid based on allelic data. Bootstrap analysis with 10,000 iterations was used to calculate the dissimilarity matrices. Using the option to alter 13 axes, the default axis as decided by the principal coordinate analysis (PCoA) was chosen to set the based on dissimilarity. Using the unrooted weighted neighbour-joining strategy, we constructed trees using the computed dissimilarity matrix. Using Dissimilarity Analysis and Representation for WINDOWS (DARWin) software version 6.05 (Perrier & Jacquemoud-Collet 2006; http://darwin.cirad.fr/darwin), the dissimilarity matrix, bootstrapping, PCoA, and tree construction for the sago palm accessions were carried out.

## RESULTS

**Figure 2:**
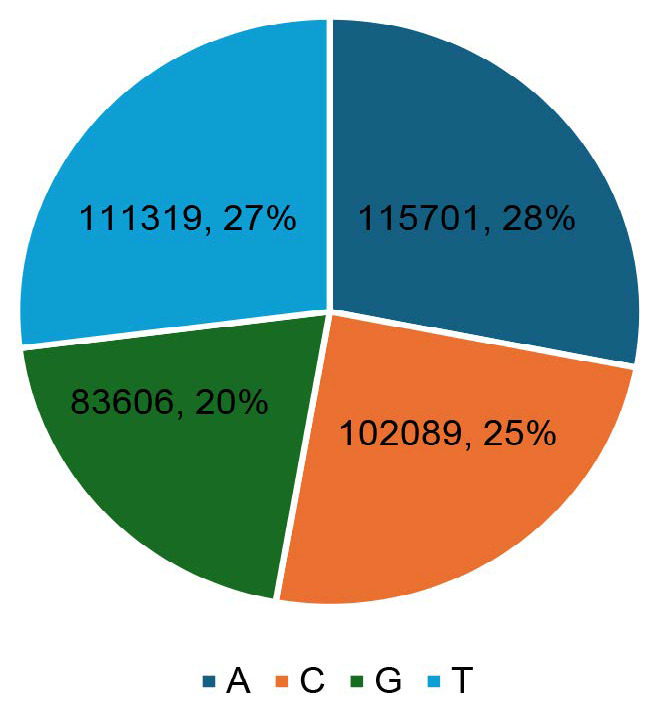
Nucleotide distribution in EST sequences of sago palms.

Regarding analysis, the total length of the EST sequence produces 412,716 bp (412 contigs) with a maximum and minimum length of 1,138 and 124 nucleotides, respectively. The results of sequence processing using the EGassembler web-based application showed 412 clean EST sequences without contaminants from vector sequences. EST sequence nucleotides were distributed with a frequency of A: 115,701 (28%), C: 102,089 (25%), G: 83,606 (20%) and T: 111,319 (27%) while the composition of GC: 185,695 (45%) (Fig. 2).

### SSR Pattern Frequency and Type

**Figure 3:**
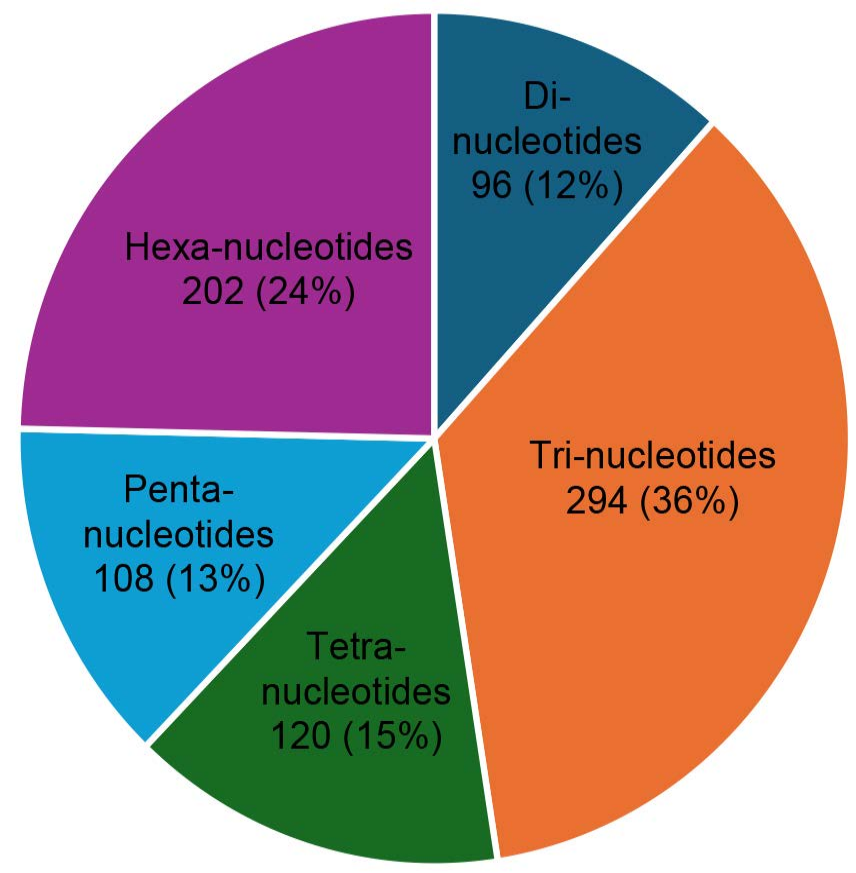
Distribution of SSR types in EST sequences of sago.

**Figure 4:**
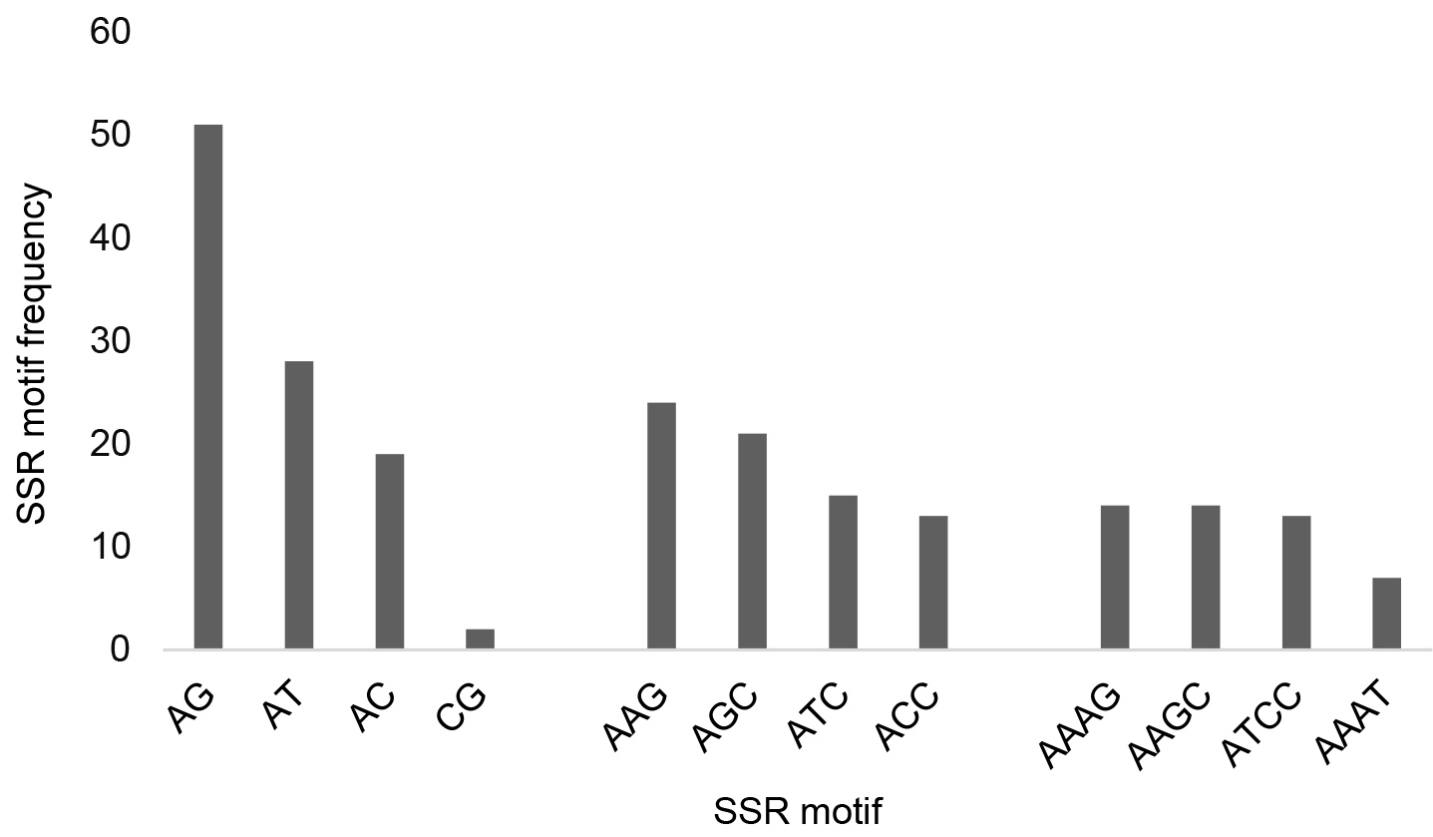
The best four SSR motif frequencies for di-, tri- and tetra-nucleotides.

A total of 820 SSRs with perfect motifs have been detected from 349 EST sequences. The frequency of SSR motifs in the EST sequence obtained from the results of this study is 1/0.5 kb of the EST sequence, or there is one SSR motif in every 0.5 kb of the EST sequence. The repeat type characterisation in EST sequences was dominated by tri-nucleotides 36% (294), followed by hexanucleotides 24% (202), tetra-nucleotides 15% (120), penta-nucleotides 13% (108) and di-nucleotides 12% (96) (Fig. 3). The highest frequency of SSR motifs in each type was AG: 49 (51%), AAG: 72 (24.5%), and AAAG: 17 (14.2%) (Fig. 4).

### Synteny Analysis

Synteny analysis was carried out using the Phytozome Online application using the BLASTn programme. From the EST sequences that were detected to have SSR and were selected, synteny analysis was carried out using *Oryza sativa* data. From the analysis, it was found that the EST sequences of sago were spread over 12 chromosomes with the percentage of similarity between 63%–100% and the e-value ranged from 0 to 0.094 (Table 2). Meanwhile, the 15 sequences that were primer designed successfully were spread on 12 rice chromosomes with a similarity percentage between 64%–100% and e-values ranging from 2.00E–19 to 0.094 (Table 2). However, there is one sequence whose synteny is not known but the primer has been successfully synthesised, namely EJK731303.

### Primer Design for EST-SSR Markers

Of the 412 EST sequences with the perfect SSR motif, 15 sequences with the SSR motif were selected. A total of 20 SSR motifs from 15 sequences allow for primer design, namely: 1 di-nucleotide, 7 tri-nucleotide, 1 penta-nucleotide and 11 hexa-nucleotides. Only 19 primer pairs of the 20 SSR motifs could be synthesised (Table 2). The primers were designed with the following criteria: optimum primer size 20 bp, melting temperature (Tm): 55°C–60°C, and GC content 45%–60%. The size of the shortest primer design product is 184 bp and the longest is 498 bp with a Tm range of 60°C and a GC value of 45%–55%.

### Sequence Annotations Containing SSR

**Figure 5:**
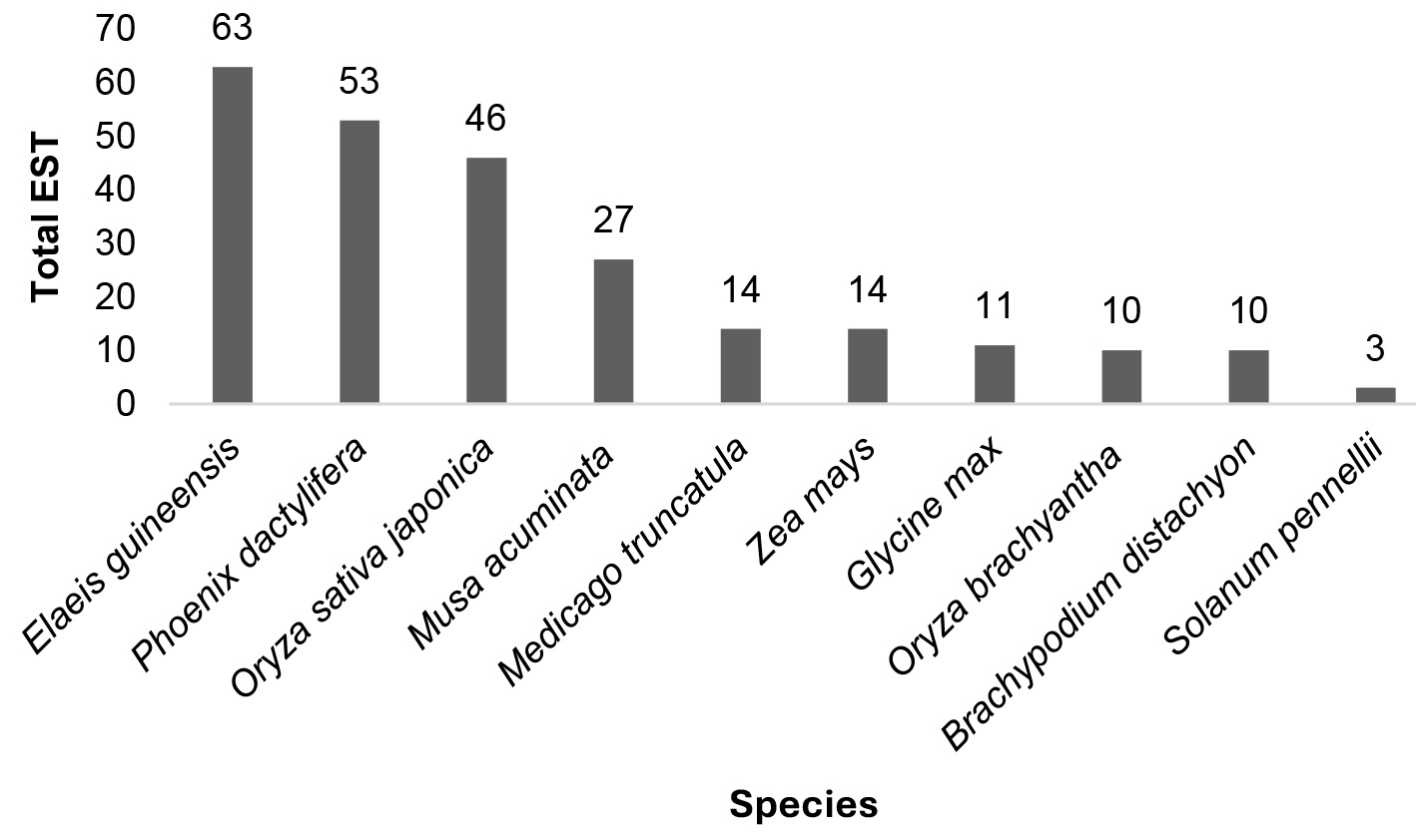
Frequency of the 10 plants with the most hits.

The primers were designed from EST sequences then analysed using BLASTx by selecting the *e*-value 0.00001 against the NCBI-nr database followed by the TAIR database (Table 3). From 15 sequences analysed, 14 sequences were identified as having a gene ontology and successfully mapped, only 1 sequence that did not have BLASTx Hit (Table 4). The 10 plant species having the highest hit frequency can be seen in Fig. 5. From these results, it can be seen that there were six monocot plants (*Elaeis guineensis*, *Phoenix dactylifera*, *Oryza sativa japonica*, *Musa acuminate*, *Zea may*s and *O. brachyantha*) and four dicot plants (*Medicago truncatula*, *Glycine max*, *Brachypodium distachyon* and *Solanum pennellii*) which have significant homology.

**Figure 6:**
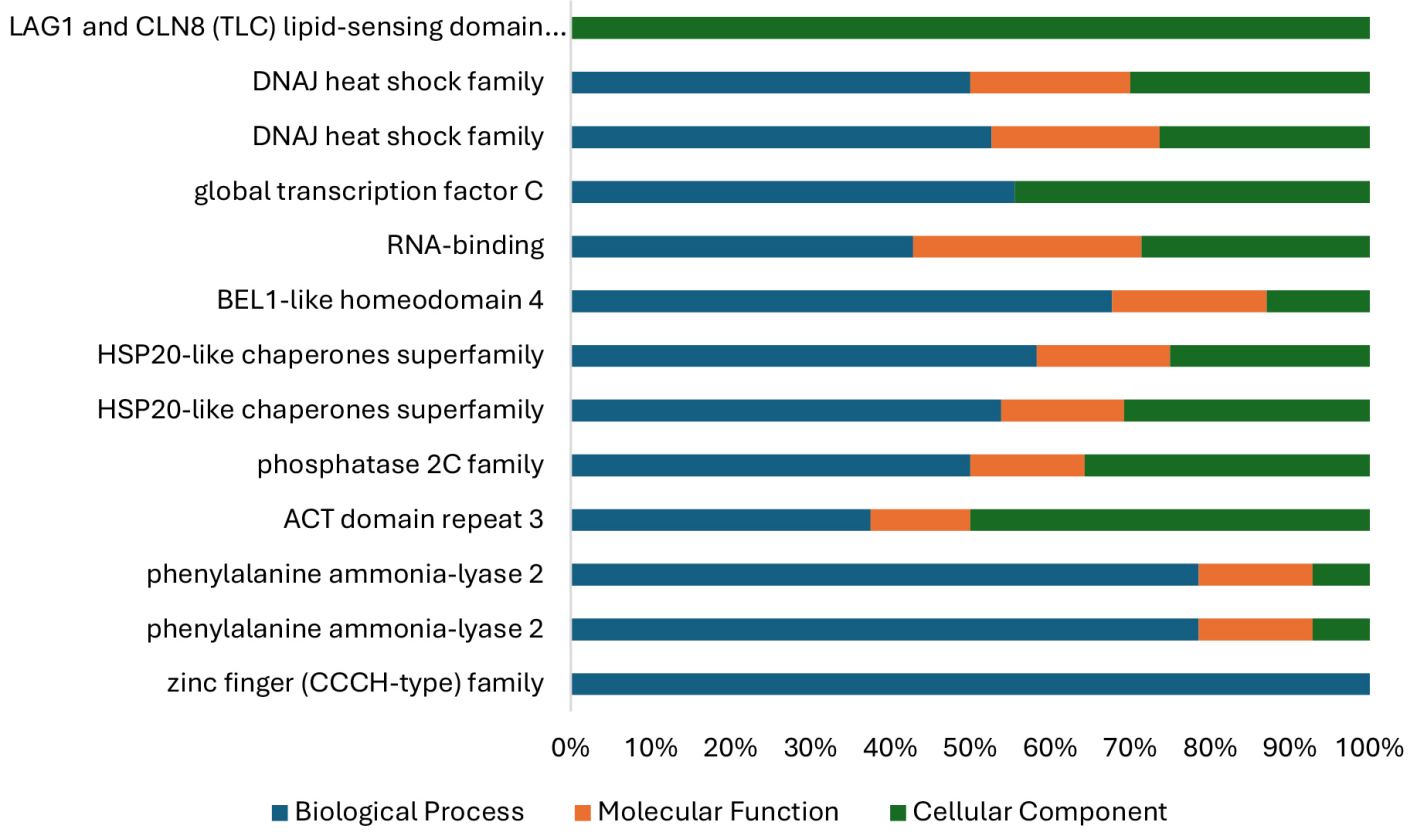
Gene ontology (GO) classification annotated for the sequence containing SSR in the cellular component, molecular function and biological processes.

The BLASTx programme on TAIR was used to search for gene annotations. The results of gene ontology annotations and functional categories based on locus identification can be seen in Fig. 6. Based on the results of the sequence annotations, a total of 290 gene ontologies can be determined and distributed into three categories: molecular functions (71), biological processes (210), and cellular components (53). The molecular function is dominated by nucleotide-binding subcategory about 19.7%, biological process subcategory is dominated by metabolic process subcategory about 62.7%, and cellular components are dominated by membrane subcategory about 62.7%.

### Genetic Relationship and Cluster Analysis

**Figure 7:**
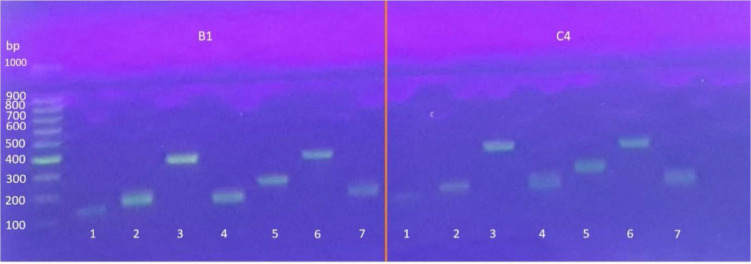
Validation of seven primers on two accessions of sago with 1% agarose gel (1–7: primer).

In this study, a total of 19 primer pairs were designed and from 15 selected sequences containing SSR motifs, 7 class I primers were synthesised (Table 2), used for validation and polymorphism assessment among 2 accessions of *M. sagu* (B1 and C4) of which 7 showed amplification and 7 were found to be polymorphic (Fig. 7).

A total of 7 SSR markers were found to be polymorphic in 21 alleles of *M. sagu* with an average number of 3 alleles per locus. The PIC values were found to range from 0.132 in the primary (EJK 731600-1 and EJK 731391-2) to 0.580 in (EJK 731455), with a mean value of 0.315. The highest (0.680) and the lowest (0.148) expected heterozygosity values were obtained with primers (EJK 731455) and (EJK 731600-1 and EJK 731391-2), respectively, with a mean value of 0.372. The range for the observed heterozygosity (Ho) was 0.154 to 0.769 with a mean value of 0.341 (Table 5).

**Figure 8:**
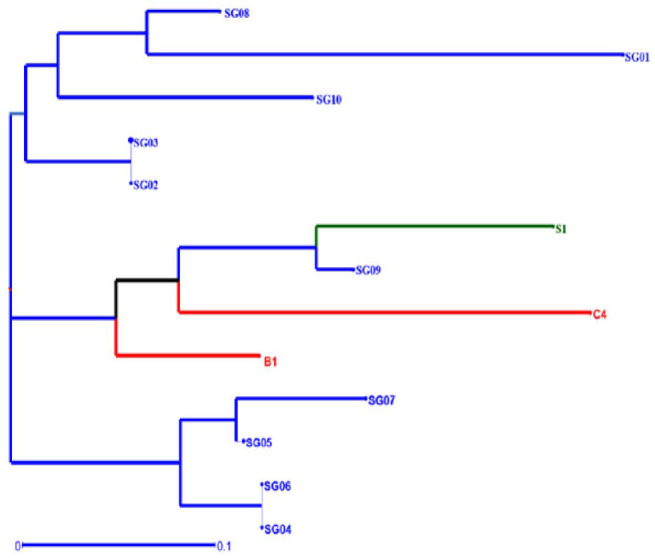
Unrooted weighted neighbour-joining cluster analysis of genetic dissimilarity as measured using amplified simple sequence repeat (SSR) markers. Blue: West Borneo accessions; Green: Java accession; Red: Sumatera and Maluku accessions.

**Figure 9:**
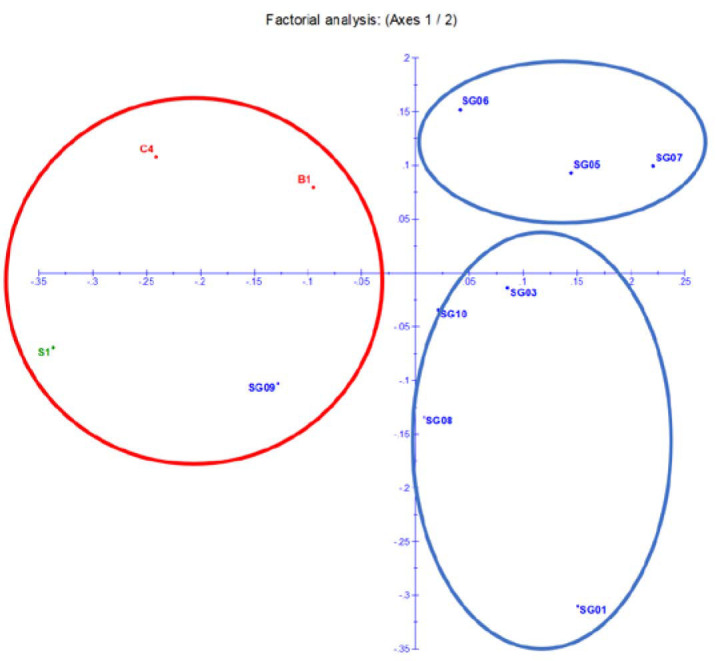
Factorial analysis based on Eigen values calculated from seven SSR markers.

Unrooted weighted neighbour-joining cluster analysis was constructed to measure genetic diversity and interrelationships between accessions in 13 accessions grouped into three large groups using Darwin software. Cluster I consist of five accessions including SG01, SG02, SG03, SG08 and SG10. Cluster II consists of four accessions S1, SG09, C4 and B1. Cluster III consists of four accessions SG04, SG05, SG06 and SG07 (Fig. 8). The dendrogram grouping classifying various accessions of *M. sagu* based on response to EST-SSR markers is the first report to the authors’ knowledge. In a previous study, Purwoko *et al*. (2019) also succeeded in grouping various accessions of *M. sagu* from various islands in Indonesia. The results of PCoA (Fig. 9) presented a two-dimensional graphical view of the genetic diversity of 13 sago palm accessions originating from four regions in Indonesia. The results observed in the PCoA were in agreement with the cluster analysis.

## DISCUSSION

Publicly available EST data have proven to be useful in the identification and development of SSR molecular markers. The EST sequences play a useful role in the establishment of markers, transcriptomic profiling, proteomic research, and gene discovery (Haq *et al*. 2021). The EST-SSR marker has advantages because it contains candidate genes and can produce molecular markers associated with certain traits (Kalia *et al*. 2011). According to Haq *et al*. (2014) and Singh *et al*. (2019), the EST-SSR marker itself is a functional molecular marker to characterise “a presumed function or a particular gene encoding enzymatic activity” that aids in numerous genomic applications in plants. According to research by Pashley *et al*. (2006), Ellis and Burke (2007) and Haq *et al*. (2014), the presence of SSRs in the expressed area or ESTs is more preserved, significant, and transferable across taxonomic boundaries than anonymous SSRs. In numerous analyses of plant genomes, including those that evaluate genetic polymorphism, genetic diversity, population genetics, biodiversity, high-resolution genetic maps, gene mapping, quantitative trait loci, germplasm characterisation, cultivar identification, paternity analysis, marker-assisted breeding taxonomy, and comparative genomic studies, EST-SSR is the preferred molecular marker (Kantety *et al*. 2002; Eujayl *et al*. 2004; Varshney *et al*. 2007; Ukoskit *et al*. 2018; Haq *et al*. 2021). EST markers were also known to originate from genomic regions that can be transcribed and conserved across multiple genomes over a wider range than other markers (Pashley *et al*. 2006).

In this study, 820 SSRs were found from 412 EST sequences of *M. sagu* or 1/0.5 kb of the EST sequence to find EST-SSR markers. This result is much lower than previous studies on the sago genome, namely 132.57/Mb (Purwoko *et al*. 2019). We found that the trinucleotide repeat sequence had a dominant frequency (36%) compared to the others. A similar situation was previously reported in *C. longa* (Purwoko *et al*. 2021), *P. violascens* (Cai *et al*. 2019), and same results were also obtained in date palm ESTs which stated that tri-nucleotides predominated from other motifs (Zhao *et al*. 2013). Dissimilar things were reported in oil palm plants that di-nucleotides predominated compared to others (Singh *et al*. 2008). However, the type of dinucleotide motif found to be the most common SSR was AG (51%) followed by AAG (24.5%) then AAAG (14.2%). This is similar to motifs in *M. sagu* genome, in which the AG and AAG motifs are predominant (Purwoko *et al*. 2019).

Some SSRs do not produce primers because of their impossible position, at the beginning or end of the sequence. According to Kale *et al*. (2012), the failure of the design of primer was due to not obtaining a suitable clamping sequence or an impossible melting temperature constraint. Primer validation is carried out to determine the ability of the primer that has been designed to be amplified or not. The primer ability to produce amplification products is influenced by several characters, such as internal stability, melting temperature, secondary structure, or competition between primers (Sint *et al*. 2012).

For annotation analysis, EST sequences with SSR and having a primer (15 sequences) were performed comparative analysis with the publicly available databases NCBI-nr and TAIR and resulted annotations for 14 (93.33%) sequences. The interesting thing is the highest hits were obtained on *Elaeis guineensis* and *Phoenix dactylifera* which are palms, making it possible that the primers synthesised from sago EST could also be used in these two plants for tranferable genotyping study across palms genera. Transferability of SSR markers indicates whether the markers are applicable to comparative mapping studies in plants (Endo *et al*. 2017). Comparative analysis with TAIR yielded 7,062 functional characteristic hits.

Our findings support the usefulness of EST-SSR markers for sago cultivar differentiation and genetic diversity and grouping analysis. Additionally, we have demonstrated the value of the created EST-SSR marker in examining the genetic diversity of the sago plant. Given that gene function is frequently established (Parida *et al*. 2009), the use of DNA coding regions for the construction of SSR is a further benefit in genetic associations (Feingold *et al*. 2005) and linkage analysis. Recently constructed EST-SSR markers have been successfully used to study association mapping for traits of interest in various commodities such as *Syringa oblata* (Yang *et al*. 2020) and *Hibiscus cannabinus* (An *et al*. 2023).

## CONCLUSION

The results of the current study demonstrate the successful identification and development of SSR markers in sago palms based on in silico EST data. A computational-based approach was used to develop and identify SSR markers from a publicly available EST database, which were further validated through a wet lab. The development of markers from DNA coding regions has a great advantage because previously known gene functions can assist in exploiting markers for specific traits. The resulting EST-SSR marker was successfully used to evaluate the genetic diversity of sago palms. In the future, the EST-SSR marker will be useful for the conservation and breeding activities of the underutilised carbohydrate-producing plants.

## Figures and Tables

**Table 1:** Parameters for designing primer.

No.	Criteria	Minimum	Maximum	Optimum
1	Amplicon size (bp)	100	500	–
2	Primer size (bp)	18	20	20
3	Melting temperature (°C)	55	60	55
4	G/C content (%)	45	60	–

**Table 2:** Description of the primers that were successfully synthesised.

No	Primer ID	Primer sequence (5′-3′)	SSR motif	Product size (bp)	*Oryza sativa* chromosome	e-value
1	EJK731455	F: GCCGACTTTCTCAGCTTGTT R: TAAGTTGCAGGGGCTTTCTC	AGGCCG	405	Chr5	2.00E–19
2	EJK731303	F: TCCAGCCTTTTCCCAACTAA R: AAGTCATGCCCATCATGTCA	AAAAAT	468	unknown	unknown
3	EJK731212	F: TGGTGGTTGACGTTGATGAT R: TAATGCAAGGGTGAGGCTTT	AACCCT	222	Chr1,2,3,5,6,10,11,12	2.00E–06
4	EJK731600	F: TCGCAGATAGCATCGAACAC R: ATCGATCGCGAGTACGTCTT	AAGGAG	374	Chr2,4,5,8,11,12	2.00E–06
5	EJK731455	F: TTCAGCTCATCCCCTTGAAT R: ATCGCTTGGTCTCGATCATT	AGC	231	Chr5	2.00E–19
6	EJK731391-1	F: ACCTCCTCCCTCACAAACCT R: AGAGGCTGTGGAGATCCTGA	AAGGAG	498	Chr2,4,5,8,11,12	2.00E–06
7	EJK731204	F: GTGCTGCTCACTGTCTCAGG R: CATGGAACAGTCCACACTGG	AATTCC	413	Chr3,4,8,9	0.047
8	EJK731454	F: ATCAGGAACACGGGACTTTG R: ACCAAGGGTATGAGCCCTCT	ATC	257	Chr1,4,10,11,12	0.052
9	EJK731329	F: GGCTTCTTGGCCTCTTCTTT R: GCAACATCTTCGACCCTTTC	ACCTCC	421	Chr1,2,3,4,7,8,9,11	0.035
10	EJK731206	F: GAACAACTGGCACCAAGGAT R: ATGTCTTCAAAGCCGACCTC	ACCTCC	357	Chr1,2,3,4,6,7,8,9	0.094
11	EJK731238	F: GACCCATGGCTTAGAACCAG R: GAGATCCTCCCGAAGAAGGT	ACACC	436	Chr2,4	0.04
12	EJK731600-1	F: GGAACATTGCAGGGTCCTTA R: GCTTTCGAAGAGGAGCTGAA	AGC	461	Chr2,4,5,8,11,12	2.00E–06
13	EJK731418	F: CCACCAGAATCTCAGTGGAA R: CCAAGACCCAACAACCACTT	AAGATG	329	Chr2,3,4	2.00E–07
14	EJK731600-2	F: GGAACATTGCAGGGTCCTTA R: TTCAACAAGGTGTTCGATGC	AAG	269	Chr2,4,5,8,11,12	2.00E–06
15	EJK731391-2	F: GGAACATTGCAGGGTCCTTA R: AGGTTTGTGAGGGAGGAGGT	AGC	338	Chr2,4,5,8,11,12	2.00E–06
16	EJK731391-3	F: GGAACATTGCAGGGTCCTTA R: TCGAAGAGGAGCTGAAGAGC	AAG	457	Chr2,4,5,8,11,12	2.00E–06
17	EJK731557	F: GCAGCAGCCAAAATAACTCC R: TGGAGCTGGATGAGTGTGAG	AAGATG	184	Chr1,2,3,4,8	0.052
18	EJK731197	F: AGGCATGATGGTCCTGAACT R: AGGATGGAGGATTGAGACGA	AG	442	Chr7,8,10,11,12	0.043
19	EJK731203	F: TCAGCCGCTGCATATGTTAC R:GCAGAGCTTCTTGGATGGTC	ATC	237	Chr2,4,5,6,8,10,12	0.051

**Table 3:** Distribution of contig sequences from Blast2Go analysis.

Sequence analysis criteria	Number of sequences
Analysed with Blast2Go	15
Ontology genes	14
BLASTx Hit	14
Non-BLASTx Hit	1
Mapped	14
The most species hits *Elaeis guineensis*	63

**Table 4:** Annotatability statistics for sequences containing SSR.

Sequence ID	Description	Length	BLAST Hit	e-value	sim mean	GO
JK731455	Zinc finger (CCCH-type) family	1,044	4	2.11E–22	52.89	1
JK731391	Phenylalanine ammonia-lyase 2	1,062	4	9.46E–149	87.30	14
JK731600	Phenylalanine ammonia-lyase 2	972	4	6.83E–121	85.28	14
JK731204	ACT domain repeat 3	967	10	4.90E–97	70.23	8
JK731203	Phosphatase 2C family	1,052	20	1.47E–109	59.08	14
JK731206	HSP20-like chaperones superfamily	569	16	5.99E–44	70.10	13
JK731329	HSP20-like chaperones superfamily	724	15	5.45E–56	68.29	12
JK731212	BEL1-like homeodomain 4	916	12	2.46E–26	80.05	31
JK731197	RNA-binding	889	6	1.95E–11	81.79	7
JK731454	Global transcription factor C	1,066	2	1.71E–49	82.40	9
JK731557	DNAJ heat shock family	1,077	20	2.02E–135	57.45	19
JK731418	DNAJ heat shock family	1,063	20	1.69E–121	58.25	20
JK731303	LAG1 and CLN8 (TLC) lipid-sensing domain containing	1,049	1	1.06E–10	83.72	1
JK731238	NA	822				

**Table 5:** Summary of observed allele number (N), polymorphism information content (PIC), observed and expected heterozygosity (Ho and He) for 13 sago palm accession.

No	SSR loci ID	Estimated allele size (bp)	N	Ho	He	PIC
1	EJK 731455	150–200	3	0.385	0.68	0.58
2	EJK 731454	200–250	3	0.769	0.591	0.472
3	EJK 731600-1	400–500	2	0.154	0.148	0.132
4	EJK 731600-2	200–290	2	0.231	0.212	0.183
5	EJK 731391-2	290–350	2	0.154	0.148	0.132
6	EJK 731391-3	400–500	3	0.231	0.342	0.303
7	EJK 731203	240–300	3	0.462	0.48	0.404
	Average		3	0.341	0.372	0.315

## References

[b1-tlsr_35-1-13] Aberlenc-Bertossi F, Castillo K, Tranchant-Dubreuil C, Chérif E, Ballardini M, Abdoulkader S, Gros-Balthazard M, Chabrillange N, Santoni S, Mercuri A, Pintaud J-C (2014). In silico mining of microsatellites in coding sequences of the date palm (Arecaceae) genome, characterization, and transferability. Applications in Plant Sciences.

[b2-tlsr_35-1-13] Amiteye S (2021). Basic concepts and methodologies of DNA marker systems in plant molecular breeding. Heliyon.

[b3-tlsr_35-1-13] An X, Liu Q, Ying J, Wei J, Dong G, Luo X, Li W, Liu T, Zhou H, Zou L, Chen C (2023). Development of expressed sequence tag–simple sequence repeat markers related to the salt-stress response of Kenaf (*Hibiscus cannabinus*). Agronomy.

[b4-tlsr_35-1-13] Aslanbay Guler B, Imamoglu E (2023). Molecular marker technologies in food plant genetic diversity studies: An overview. Foods and Raw Materials.

[b5-tlsr_35-1-13] Cai K, Zhu L, Zhang K, Li L, Zhao Z, Zeng W, Lin X (2019). Development and characterization of EST-SSR markers from RNA-seq data in *Phyllostachys violascens*. Frontiers in Plant Science.

[b6-tlsr_35-1-13] D’Imperio M, Viscosi V, Scarano MT, D’Andrea M (2011). Integration between molecular and morphological markers for the exploitation of olive germplasm (*Olea europaea*). Science Horticulture.

[b7-tlsr_35-1-13] Duran C, Singhania R, Raman H, Batley J, Edwards D (2013). Predicting polymorphic EST-SSRs in silico. Molecular Ecology Resources.

[b8-tlsr_35-1-13] Ellis JR, Burke JM (2007). EST-SSRs as a resource for population genetic analyses. Heredity.

[b9-tlsr_35-1-13] Endo C, Yamamoto N, Kobayashi M, Nakamura Y, Yokoyama K (2017). Development of simple sequence repeat markers in the halophytic turf grass *Sporobolus virginicus* and transferable genotyping across multiple grass genera/species/genotypes. Euphytica.

[b10-tlsr_35-1-13] Eujayl I, Sledge MK, Wang L, May GD, Chekhovskiy K, Zwonitzer JC, Mian MA (2004). *Medicago truncatula* EST-SSRs reveal cross-species genetic markers for Medicago spp. Theoretical and Applied Genetics.

[b11-tlsr_35-1-13] Feingold S, Lloyd J, Norero N, Bonierbale M, Lorenzen J (2005). Mapping and characterization of new EST-derived microsatellites for potato (*Solanum tuberosum* L.). Theoretical and Applied Genetics (Theoretische und angewandte Genetik).

[b12-tlsr_35-1-13] Flach M (1995). Research priorities for sago palm development in Indonesia and Sarawak: An agenda for research. ISHS Acta Horticulturae.

[b13-tlsr_35-1-13] Gotz S, Garcia-Gomez JM, Terol J, Williams TD, Nagaraj SH, Nueda MJ, Robles M, Talon M, Dopazo J, Conesa A (2008). High-throughput functional annotation and data mining with the Blast2GO suite. Nucleic Acids Research.

[b14-tlsr_35-1-13] Hailu G, Asfere Y (2020). The role of molecular markers in crop improvement and plant breeding programs: A review. Agricultural Journal.

[b15-tlsr_35-1-13] Haq SU, Dhingra P, Sharma M, Kothari SL, Kachhwaha S (2021). Plasticity of tandem repeats in expressed sequence tags of angiospermic and nonangiospermic species: Insight into cladistic, phenetic and elementary explorations. Journal of Applied Biology and Biotechnology.

[b16-tlsr_35-1-13] Haq SU, Jain R, Sharma M, Kachhwaha S, Kothari SL (2014). Identification and characterization of microsatellites in expressed sequence tags and their cross transferability in different plants. International Journal of Genomics.

[b17-tlsr_35-1-13] Jain N, Patil GB, Bhargava P, Nadgauda RS (2014). In silico mining of EST-SSRs in *Jatropha curcas* L. towards assessing genetic polymorphism and marker development for selection of high oil yielding clones. American Journal of Plant Sciences.

[b18-tlsr_35-1-13] Jong FS (1995). Research for the development of sago palm (Metroxylon sagu Rottb.) cultivation in Sarawak, Malaysia.

[b19-tlsr_35-1-13] Kale SM, Pardeshi VC, Kadoo NY, Ghorpade PB, Jana MM, Gupta VS (2012). Development of genomic simple sequence repeat markers for linseed using next generation sequencing technology. Moleculer Breeding.

[b20-tlsr_35-1-13] Kalia RK, Rai MK, Kalia S, Singh R, Dhawan AK (2011). Microsatellite markers: An overview of the recent progress in plants. Euphytica.

[b21-tlsr_35-1-13] Kantety RV, La Rota M, Matthews DE, Sorrells ME (2002). Data mining for simple sequence repeats in expressed sequence tags from barley, maize, rice, sorghum and wheat. Plant Molecular Biology.

[b22-tlsr_35-1-13] Kumar P, Gupta VK, Misra AK, Modi DR, Pandey BK (2009). Potential of molecular markers in plant biotechnology. Plant Omics Journal.

[b23-tlsr_35-1-13] Maskromo I, Larekeng SH, Novarianto H, Sudarsono S (2016). Xenia negatively affecting kopyor nut yield in Kalianda Tall Kopyor and Pati Dwarf Kopyor coconuts. Emirates Journal of Food and Agriculture.

[b24-tlsr_35-1-13] Mayer C, Leese F, Tollrian R (2010). Genome-wide analysis of tandem repeats in *Daphnia pulex*: A comparative approach. BMC Genomics.

[b25-tlsr_35-1-13] Molla MR, Islam MN, Rohman MM, Rahman L (2010). Microsatellite allele size profiling to determine varietal identity and genetic diversity among groundnut varieties in Bangladesh. Natural Sciences.

[b26-tlsr_35-1-13] Mondini L, Noorani A, Pagnotta MA (2009). Assessing plant genetic diversity by molecular tools. Diversity.

[b27-tlsr_35-1-13] Nejad AM, Tonomura K, Kawashima S, Moriya Y, Suzuki M, Itoh M, Kanehisa M, Endo T, Goto S (2006). EGassembler: Online bioinformatics service for large-scale processing, clustering and assembling ESTs and genomic DNA fragments. Nucleic Acids Research.

[b28-tlsr_35-1-13] Novero AU, Ma BM, Hannah JE (2012). Epigenetic inheritance of spine formation in sago palm (*Metroxylon sagu* Roettb.). Plant Omics Journal.

[b29-tlsr_35-1-13] Parida SK, Kalia SK, Kaul S, Dalal V, Hemaprabha G, Selvi A, Pandit A, Singh A, Gaikwad K, Sharma TR, Srivastava PS, Singh NK, Mohapatra T (2009). Informative genomic microsatellite markers for efficient genotyping applications in sugarcane. TAG Theoretical and Applied Genetics (Theoretische und angewandte Genetik).

[b30-tlsr_35-1-13] Pashley CH, Ellis JR, McCauley DE, Burke JM (2006). EST databases as a source for molecular markers: Lessons from Helianthus. Journal of Heredity.

[b31-tlsr_35-1-13] Perrier X, Jacquemoud-Collet JP (2006). DARWin software.

[b32-tlsr_35-1-13] Pesik A, Efendi D, Novarianto H, Dinarti M, Maskromo I, Tenda ET, Sudarsono S (2015). Keragaman dan hubungan genetik antara kelapa tetua genjah kuning nias. Buletin Palma.

[b33-tlsr_35-1-13] Pesik A, Efendi D, Novarianto H, Dinarti D, Sudarsono S (2017). Development of SNAP markers based on nucleotide variability of WRKY genes in coconut and their validation using multiplex PCR. Biodiversitas Journal of Biological Diversity.

[b34-tlsr_35-1-13] Priyanka P, Kumar D, Yadav A, Yadav K, Dwivedi U (2017). Analysis of simple sequence repeats information from floral expressed sequence tags resources of papaya (*Carica papaya* L.). American Journal of Plant Sciences.

[b35-tlsr_35-1-13] Purwoko D, Cartealy IC, Tajuddin T, Dinarti D, Sudarsono S (2019). SSR identification and marker development for sago palm based on NGS genome data. Breeding Science.

[b36-tlsr_35-1-13] Purwoko D, Zulaeha S, Tajuddin T, Khairiyah H, Fauzi RZ, Priyanti (2021). SSR markers characterization for Temu Ireng (*Curcuma aeruginosa* Roxb.) generated from EST of *Curcuma longa*. Jurnal Bioteknologi dan Biosains Indonesia.

[b37-tlsr_35-1-13] Salgotra RK, Chauhan BS (2023). Genetic diversity, conservation, and utilization of plant genetic resources. Genes.

[b38-tlsr_35-1-13] Singh R, Zaki NM, Ting NC, Rosli R, Tan SG, Low ETL, Ithnin M, Cheah SC (2008). Exploiting an oil palm EST database for the development of gene-derived SSR markers and their exploitation for assessment of genetic diversity. Biologia.

[b39-tlsr_35-1-13] Singh RB, Singh B, Singh RK (2019). Development of potential dbEST-derived microsatellite markers for genetic evaluation of sugarcane and related cereal grasses. Industrial Crops and Products.

[b40-tlsr_35-1-13] Sint D, Raso L, Traugott M (2012). Advances in multiplex PCR: Balancing primer efficiencies and improving detection success. Methods in Ecology and Evolution.

[b41-tlsr_35-1-13] Supek F, Bošnjak M, Škunca N, Šmuc T (2011). REVIGO summarizes and visualizes long lists of gene ontology terms. PLoS ONE.

[b42-tlsr_35-1-13] Tinche, Asmono D, Dinarty D, Sudarsono S (2014). Genetic diversity oil palm (*Elaeis guineensis* Jacq.) Nigeria population based on SSR (Simple Sequence Repeats) marker analysis. Buletin Palma.

[b43-tlsr_35-1-13] Ukoskit K, Posudsavang G, Pongsiripat N, Chatwachirawong P, Klomsaard P, Poomipant P, Tragoonrung S (2018). Detection and validation of EST-SSR markers associated with sugar-related traits in sugarcane using linkage and association mapping. Genomics.

[b44-tlsr_35-1-13] Untergasser A, Cutcutache I, Koressaar T, Ye J, Faircloth BC, Remm M, Rozen SG (2012). Primer3: New capabilities and interfaces. Nucleic Acids Research.

[b45-tlsr_35-1-13] Varshney RK, Marcel TC, Ramsay L, Russell J, Röder MS, Stein N, Waugh R, Langridge P, Niks RE, Graner A (2007). A high density barley microsatellite consensus map with 775 SSR loci. Theoretical and Applied Genetics.

[b46-tlsr_35-1-13] Vieira LD, da Silva JO, Pereira CCO, de Carvalho SA, Silveira RDD, Malafaia G, de Menezes IPP (2016). In silico identification of putative expressed sequence tag (EST)-simple sequence repeats (SSRs) markers of resistance to *Meloidogyne* spp. in common bean. African Journal of Agricultural Research.

[b47-tlsr_35-1-13] Yang Y, He R, Zheng J, Hu Z, Wu J, Leng P (2020). Development of EST-SSR markers and association mapping with floral traits in Syringa oblata. BMC Plant Biology.

[b48-tlsr_35-1-13] Zhao Y, Williams R, Prakash CS, He G (2013). Identification and characterization of gene-based SSR markers in date palm (*Phoenix dactylifera* L.). BMC Plant Biology.

